# Evolution of Endoscopic Spine Surgery: Contemporary Advances and Future Directions

**DOI:** 10.1007/s12178-026-10058-3

**Published:** 2026-09-25

**Authors:** Gregory Glauser, Shaarada Srivatsa, Bilal B. Butt, Mohamed Macki, Christoph P. Hofstetter, Osama N. Kashlan

**Affiliations:** 1 Department of Neurosurgery, Neurological Institute, Cleveland Clinic, Cleveland, OH USA; 2 Cleveland Clinic Lerner College of Medicine, Cleveland, OH USA; 3 Department of Neurosurgery, University of Washington, Seattle, WA USA; 4 Cleveland Clinic - Hillcrest Hospital, 6780 Mayfield Rd, Mayfield Heights, OH 44124 USA

**Keywords:** Endoscopic fusion, Endoscopic spine surgery, Minimally invasive spine surgery, Lumbar decompression

## Abstract

**Purpose of review:**

Endoscopic spine surgery has evolved from non-visualized percutaneous discectomy only, into a comprehensive minimally invasive surgical platform with expanding indications across cervical, thoracic, and lumbar pathology. This review examines endoscopic spine surgery’s historical evolution, summarizes recent advances in technology and clinical application, and evaluates current evidence regarding outcomes, complications, and future directions.

**Recent findings:**

Endoscopic spine surgery demonstrates clinical outcomes comparable to traditional open and minimally invasive approaches for lumbar disc herniation and spinal stenosis. Advantages of endoscopic approach include reduced tissue disruption, decreased blood loss, shorter hospitalization, expedited recovery, and faster return to work. Emerging applications include endoscopic-assisted fusion procedures, outpatient spine surgery pathways, and revision decompression techniques. Recent meta-analyses additionally suggest comparable outcomes between uniportal and biportal approaches, although differences in visualization, ergonomics, and learning curves remain areas of ongoing investigation.

**Summary:**

Current data supports the safety and efficacy of endoscopic surgery in appropriately selected patients, particularly for lumbar decompression procedures. Barriers including steep learning curves, limited formalized training pathways, equipment costs, reimbursement challenges, and the need for long-term comparative outcomes data continue to limit widespread adoption. Future integration of robotics, artificial intelligence, navigation technologies, and biologic augmentation may further expand the role of endoscopic spine surgery.

## Introduction

Degenerative spinal disorders represent a leading cause of disability worldwide, with substantial healthcare utilization and societal cost [1]. The prevalence of degenerative spine pathology is expected to grow, with reports suggesting roughly 90% of individuals 60 years of age or older have degenerative changes in imaging studies [2]. Over the past few decades, surgical management of spinal pathologies has shifted toward techniques that minimize tissue disruption while maintaining post-operative outcomes [3]. Endoscopic spine surgery embodies this paradigm shift, with a minimally invasive approach that utilizes direct visualization through small working channels, to achieve decompression of the neural elements.

Contrary to traditional open approaches, endoscopic surgery is predicated on targeted pathology treatment. Access to pathology is attained through natural anatomic corridors, which minimize collateral damage to the paraspinal musculature, facet joints and critical load bearing bony structures [4]. While early iterations were limited in adoption and scope, technological advancements and growing clinical evidence have catalyzed widespread interest in endoscopic surgery. This article evaluates the evolution of endoscopic spine surgery, highlighting key milestones, current applications and future opportunities.

## Historical Foundations

Endoscopic spine surgery has evolved gradually, in conjunction with technological advances, over the past century. Arthroscopic instruments were first used to visualize the spinal cord and nerve roots in cadavers during the 1930s, with the first human “myeloscopy” procedures described in 1938 [5].

### First Generation: Early Endoscopic Discectomy

**Fig. 1:**
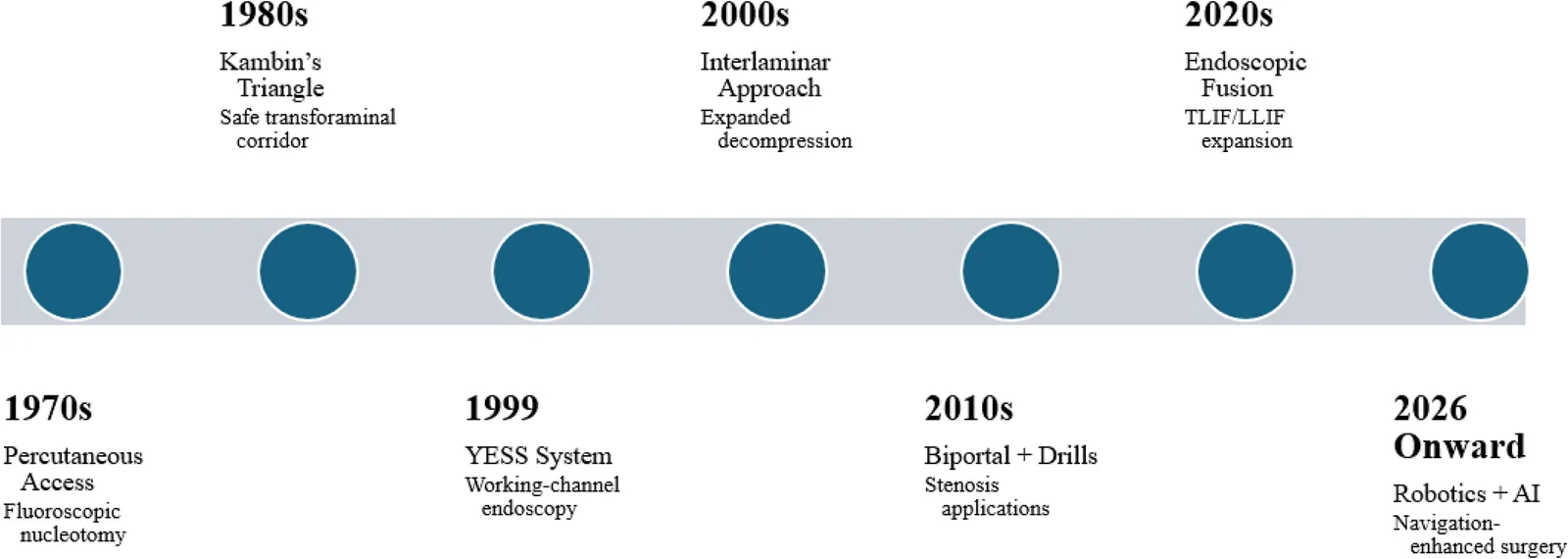
Evolution of Endoscopic Spine Surgery Timeline

Percutaneous approaches to access the disc space under fluoroscopic guidance originated in the 1970s (Fig. 1) [5]. Initial percutaneous spine surgeries were for lumbar discectomies through cannular based approaches [6, 7]. The next breakthrough was the anatomic description of Kambin’s triangle, which detailed the safe triangular zone for addressing foraminal pathology, thus facilitating the transforaminal approach [8]. Subsequently, Schreiber et al. and Kambin et al. published series of discectomies completed under direct visualization using endoscopes [9, 10].

The initial foray into endoscopic spine surgery was catalyzed by the transforaminal approach. In 1999, the Yeung Endoscopic Spine System (YESS) was introduced, which supplanted the existing percutaneous discectomy [11]. Fiberoptic visualization prior to the YESS allowed surgeons to directly observe herniated disc material intra-operatively. However, early endoscopic systems were constrained by limited image quality and instrumentation [11]. YESS enabled working-channel endoscopy with dedicated instruments and improved optics. During this period, discectomies were performed as intra-discal, or “inside-out” procedures. While YESS was a pivotal innovation in endoscopic spine surgery, adoption remained limited due to technical challenges, steep learning curves and skepticism about its effectiveness compared to traditional approaches [11].

### Second and Third Generations: Technique Refinement and Growing Indications

Due to a combination of anatomic limitations and prolonged operative/fluoroscopy time with the transforaminal approach, the 2000s saw the next innovation in endoscopic spine surgery – the interlaminar approach [11]. Interlaminar approaches provide similar visualization to traditional open surgery, with a similar technique to tubular approaches. The interlaminar approach is particularly advantageous for lower lumbar levels, which have large interlaminar windows. Further, the interlaminar approach enabled surgeons to access central and paracentral disc herniations, which were anatomically challenging from a transforaminal approach [12].

This period also saw growing adoption of the biportal technique, after the introduction of the endoscopic drill in 2008 [13]. Endoscopic drills and multiple access ports allowed for further expansion of endoscopic indications. Endoscopic surgery for degenerative lumbar stenosis gained continued popularity during the 2010s, with advantages of less bone resection and less muscle damage in comparison to traditional approaches [11].

## Current State and Recent Advances

### Advanced Applications

**Fig. 2:**
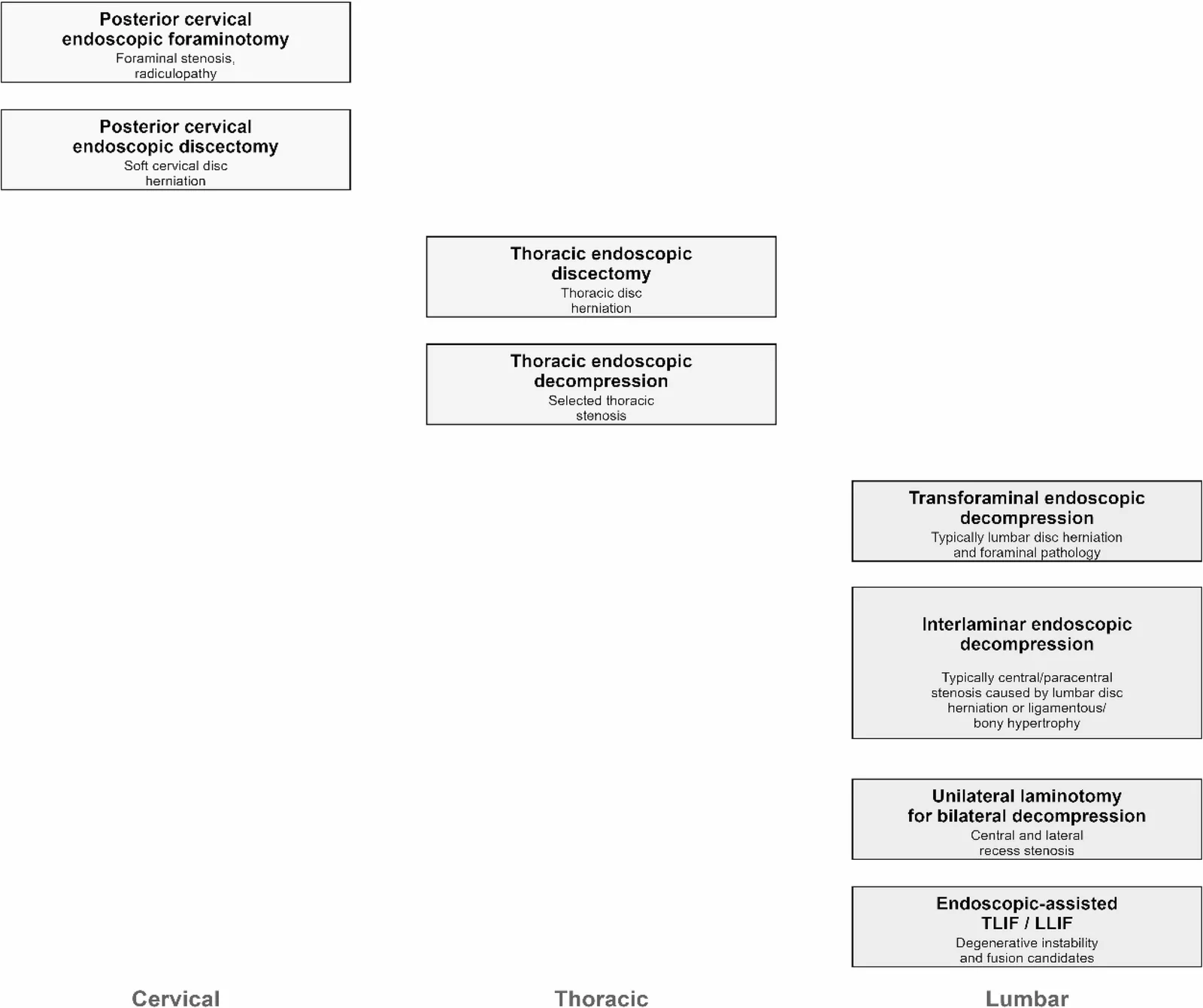
Applications of Endoscopic Spine Surgery by Region and Indication

Endoscopic spine surgery has expanded substantially beyond its initial niche of lumbar disc herniation. Current applications include decompression of central, lateral recess, and foraminal stenosis; bilateral decompression through unilateral approaches; and selected cervical and thoracic procedures (Fig. 2) [5]. Posterior endoscopic cervical foraminotomy (PECF) and posterior endoscopic cervical discectomy (PECD) have emerged as safe, effective, motion-sparing ways to treat cervical spine pathology [5, 14–17]. Endoscopic unilateral laminotomy for bilateral decompression has further expanded posterior cervical applications to selected cases of central canal stenosis and myelopathy. While these approaches achieve neural decompression while preserving motion, the confined cervical working corridor and proximity of the spinal cord, exiting nerve roots, and vertebral artery increase technical complexity.

Thoracic endoscopic surgery is a growing area of interest, with reported use in treating disc herniations and stenosis cases [18–20]. The potential margin of benefit from endoscopy may be particularly substantial in the thoracic spine because conventional treatment of ventral thoracic pathology often requires transpedicular, costotransversectomy, lateral extracavitary, or transthoracic exposure with greater muscle and osseous disruption and typically requires instrumented fusion. Recent comparative evidence suggests that endoscopic thoracic discectomy can achieve neurological improvement comparable to open surgery while substantially reducing operative time, blood loss, length of hospitalization, need for instrumentation, and overall cost [21–22]. This increasing difference in approach-related morbidity illustrates how the relative benefit of endoscopy becomes greater as the morbidity of the corresponding conventional exposure increases.

In recent years, another development has been the emergence of endoscopic-assisted fusion procedures. Endoscopic transforaminal lumbar interbody fusion (TLIF) and lateral lumbar interbody fusion (LLIF) have emerged as viable treatment options, with comparable clinical outcomes to their minimally invasive counterparts [23–27].

During endoscopic TLIF, decompression and facetectomy are completed through the endoscopic corridor, followed by annulotomy, discectomy and endplate preparation under direct endoscopic visualization. Bone graft is then introduced to the prepared disc space, followed by placement of a specially designed endoscopic interbody cage. Percutaneous pedicle screw placement is then completed as indicated. Endoscopic LLIF is carried out with similar principles, through a lateral retroperitoneal approach [28] A safe trajectory is localized either through or adjacent to the psoas, with a working channel endoscope used for direct visualization during the discectomy, disc space preparation and cage placement [28].

Endoscopic fusion remains in its early stages, with promising results secondary to reduced soft tissue disruption and expedited recovery. However, data surrounding long-term fusion outcomes and results of controlled trials are lacking to date [5].

### Outpatient Spine Surgery

Endoscopic spine surgery is uniquely aligned with the growing trend of outpatient spine surgery. Full-endoscopic lumbar discectomy is frequently performed under local anesthesia [29] In cases where local anesthetic alone is insufficient, intravenous sedation, epidural anesthesia, or spinal (intrathecal) anesthesia may be utilized [29]. Both approaches provide effective pain control while avoiding intubation and general anesthesia. Local anesthesia additionally preserves neurologic function intraoperatively, thereby enabling real-time awake neuromonitoring [29]. Endoscopic spine surgery often provides immediaterelief from radicular symptoms, as early as postoperative day 0. Data consistently demonstrate patient reported reductions in back and leg pain by postoperative day 1 whereas improvement in back pain–related disability and overall physical function may take up to 3 months [30]. Similarly, recovery of physical function following treatment for spinal stenosis depends largely on preoperative activity levels and may take up to 6 months to become evident [31]. Importantly, given the favorable complication profile, postoperative follow-up may be facilitated through smartphone applications [32].

The evolution of outpatient spine surgery has occurred in parallel with enhanced recovery after surgery (ERAS) protocols, which aim to optimize perioperative clinical pathways using evidence-based strategies. ERAS protocols in elective spine surgery have been associated with reduced opioid utilization, improved functional outcomes, increased rates of home discharge, and reduced blood loss [33, 34]. Literature evaluating ERAS for endoscopic spine surgery is evolving, but preliminary studies demonstrate shorter length of stay and improved post-operative pain scores.^35^.

In addition to optimized perioperative care via ERAS pathways, careful patient selection remains a critical component of outpatient spine surgery [36]. To date, the majority of outpatient spine surgical indications are lumbar stenosis and lumbar disc herniation, with most patients selected for outpatient surgery being relatively young and medically healthy [36, 37]. These advantages also align closely with value-based care models, which prioritize outcomes relative to cost. Preliminary data suggest that outpatient spine surgery may reduce healthcare spending by approximately 10% per episode, although further economic analyses remain necessary [38].

Beyond streamlining perioperative logistics, endoscopic spine surgery enables a fundamental paradigm shift in how postoperative care is delivered. Because recovery after tissue-sparing endoscopic surgery is front-loaded and unfolds predominantly outside the hospital, the clinically meaningful postoperative course is poorly captured by conventional, coordinator-dependent follow-up anchored to fixed 3- and 12-month clinic visits. Smart phone application-based digital platforms address this mismatch by shifting monitoring from the clinic to the patient: smartphone software can capture high-frequency, patient-directed patient-reported outcome measures (PROMs), support asynchronous patient–provider communication and virtual wound surveillance, and enable active, real-time care delivery during the critical first postoperative weeks. The feasibility of this model at scale was recently demonstrated by a multicenter Endoscopic Spine Research Group registry (SPINEHealthie), in which 91% of eligible patients participated, 85% completed postoperative PROMs, 67% completed at least five PROMs within the first postoperative week, and 53% submitted wound images, yielding more than 142,000 prospective data points across 1,656 patients [39]. Importantly, platform-supported care was associated with reduced clinic utilization and fewer emergency department visits, suggesting that virtual follow-up can substitute for, rather than merely supplement, traditional in-person surveillance. Together, these developments reframe endoscopic spine surgery not merely as a less invasive operative technique, but as the enabling substrate for a patient-directed, digitally integrated model of postoperative care.

## Technological Drivers of Evolution

### Visualization and Optics

**Fig. 3:**
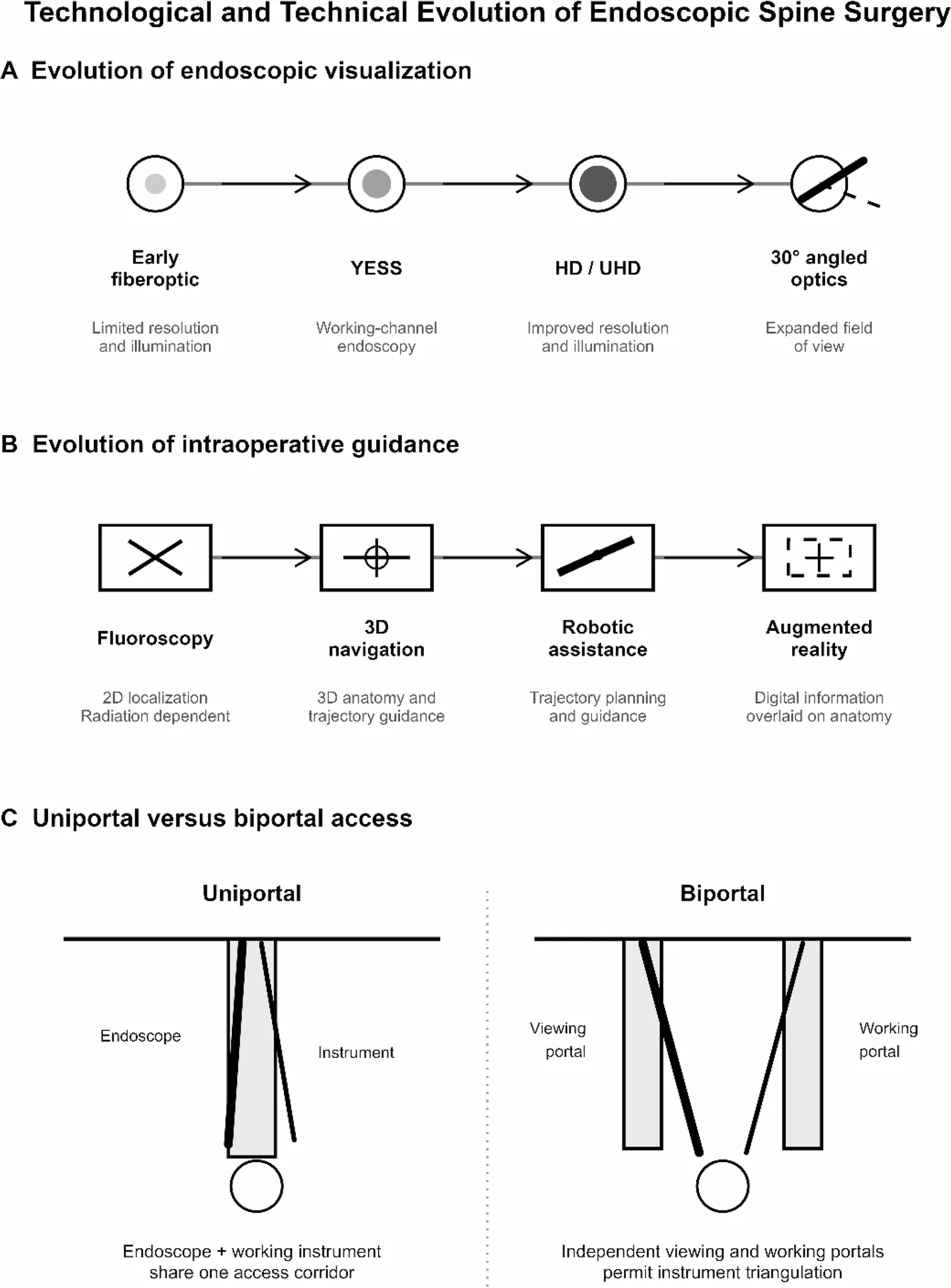
Technological and Technical Evolution of Endoscopic Spine Surgery. (**A**) Evolution of endoscopic visualization (**B**) Evolution of intraoperative localization and guidance (**C**) Schematic comparison of uniportal endoscopy, in which the endoscope and working instruments share a common working corridor, and biportal endoscopy, which uses separate viewing and working portals

Early iterations of endoscopic techniques were limited by low image resolution, poor illumination and restricted fields of view. Modern systems incorporate high-definition (HD) and ultra-HD optics, upgraded xenon and LED light sources and continuous irrigation systems. These advances collectively afford enhanced visualization in the narrow working corridors of endoscopic surgery (Fig. 3A).^5^.

Continuous irrigation not only clears the operative field of blood and debris but also facilitates tissue plane dissection and improves visualization of neural structures [7]. Furthermore, 15- and 30-degree angled endoscopes have significantly expanded the surgeon’s field of view, allowing for improved access to pathology within the lateral recess and neural foramina [8]. Improved visualization has facilitated the use of endoscopic approaches to even smaller working spaces, namely the cervical and thoracic spine. Without advances in LED illumination, ultra-HD optics, angled scopes, and irrigation systems, cervical endoscopic decompression procedures would likely not be technically feasible [5].

### Instrumentation and Biologics

Expansion of endoscopic indications would not have been possible without continued innovation in endoscopic instrumentation. Early endoscopic systems were designed for discectomy, with limited capacity to address bony pathology [5]. Modern endoscopic systems carry a wide array of instruments, including articulated graspers, bipolar and radiofrequency coagulation devices, flexible dissectors, Kerrison punches and high-speed endoscopic drills [11].

Bony removal for the treatment of central canal and foraminal stenosis was enabled by the development of high-speed diamond and burr drills. Similarly, flexible and articulated instruments have allowed for improved maneuverability within narrow working corridors, allowing for bilateral decompression and complex procedures through unilateral approaches [5]. Endoscopic reamers, expandable cages—including conforming polyethylene terephthalate mesh cages (“bag of bones”)—and other specialized interbody instrumentation now permit lumbar interbody fusion through narrow endoscopic corridors [5].

Osteobiologics and regenerative technologies designed to enhance arthrodesis have also become an important area of focus within endoscopic fusion surgery. Biologics may play a particularly important role in endoscopic fusion because reduced surgical exposure may limit graft volume compared with traditional open approaches [5]. While biomimetic technologies have shown promise in preclinical studies, the most consistently effective agent to date remains recombinant human bone morphogenetic protein-2 (rhBMP-2) [40, 41]. The integration of biologics with endoscopic fusion remains an evolving field, and long-term comparative data are limited.

### Navigation and Emerging Technologies

Navigation technologies, robotics and augmented reality have catalyzed a novel phase in the evolution of endoscopic spine surgery (Fig. 3B). Primitive endoscopic surgery relied upon fluoroscopy, which drove up radiation exposure for patients and surgical personnel. Navigation systems use computed tomography-based guidance, with resultant reduced radiation exposure, improved localization and optimized instrument trajectory during endoscopic cases [5]. Iterating upon original navigation technologies, robotic-assisted trajectory planning and instrument guidance offers further improvement in precision [42]. Robot guidance may decrease technical variability and flatten the technical learning curve for endoscopic spine surgery [42].

Augmented Reality (AR) and Artificial Intelligence (AI) represent additional burgeoning technologies which may have implications for endoscopic spine surgery. AR may enhance anatomical orientation intraoperatively through overlay visualization [43]. Further, AI applications include predictive analytics, preoperative planning and intraoperative navigation [44]. Although these technologies remain in their infancy, they reflect a broader trend within spine surgery toward increasingly technology-enhanced surgical care.

## Clinical Outcomes

### Comparative Effectiveness of Open and Endoscopic Techniques

The recent surge in adoption of endoscopic techniques has resulted in a spate of studies examining the clinical outcomes of endoscopic surgeries, both in case series format and in comparison, to open approaches (Table 1). Multiple studies have shown clinically significant functional improvement after endoscopic lumbar discectomy and decompression, demonstrating that interlaminar endoscopic discectomy (IED), transforaminal endoscopic discectomy (TED), unilateral biportal endoscopic discectomy (UBED), and microendoscopic discectomy (MED) are all safe and effective procedures to treat these pathologies [45–47]. There have also been several recent non-inferiority meta-analyses that demonstrate comparative effectiveness of open and endoscopic microdiscectomy and lumbar decompression [48]. These analyses focus mainly on patient reported outcome measures such as Oswestry Disability Index (ODI) and Visual Analog Scale (VAS), as well as traditional outcome metrics such as length of stay, complication and reoperation rate, and blood loss (EBL). A 2022 meta-analysis by Li et al. reported no significant difference in complication rate, reoperation rate, or length of stay between open discectomy and micro-endoscopic discectomy [49]. A second meta-analysis of studies comparing endoscopic discectomy and conventional open techniques found comparable VAS and ODI scores, and comparable complication and reoperation rates, but length of stay was significantly shorter in the endoscopic group [50]. A third meta-analysis reported similar results in terms of lower length of stay and comparable reoperation and complication rates, but also examined time to return to work, which was significantly shorter in the endoscopic than open group, and noted that postoperative dysesthesia was not significantly different between the two groups [15, 51]. Large data-set analyses of National Surgery Quality Improvement Program (NSQIP) data have shown similar findings, with comparable length of stay and operative time, as well as lower overall complication rates and blood transfusion rates in endoscopic cases compared to open [52, 53].

**Table 1:** Summary of clinical outcomes of endoscopic approaches separated by surgical indication

Indication	Key Clinical Outcomes	Perioperative Advantages	Complications / Limitations	Representative Evidence	Study Design
Lumbar Disc Herniation	Comparable improvement in ODI and VAS vs. microdiscectomy; high patient satisfaction; durable symptom relief	Shorter length of stay (LOS), reduced blood loss, faster return to work	Early learning curve may increase risk of dural tear, dysesthesia, incomplete decompression; recurrence rates similar to open	Rajadurai et al., Liu et al., Yong et al. [15, 45, 48]	Meta-analysis; Meta-analysis; Systematic review
Lumbar Spinal Stenosis	Significant improvement in functional outcomes; ULBD techniques show comparable decompression efficacy to open laminectomy	Reduced tissue disruption, lower postoperative pain, shorter hospitalization	Technically demanding; may be less effective in severe central stenosis or multilevel disease	Wang et al., Tornatore et al., Ward et al. [46, 47, 53]	Retrospective cohort; Systematic review
Cervical Spine (e.g., foraminal stenosis, disc herniation)	Early studies show comparable symptom relief and neurologic recovery vs. ACDF/posterior foraminotomy	Smaller incisions, faster recovery, preservation of motion (non-fusion techniques)	Limited long-term data; narrow operative corridor increases technical difficulty	Bucknall et al [66], Ruetten et al [67]	Retrospective cohort; Randomized controlled trial; Systematic review
Thoracic Spine	Feasibility demonstrated; effective for selected disc herniations and stenosis	Minimally invasive access reduces morbidity compared to open thoracotomy	Sparse data; technically challenging; risk of neural injury in confined space	Choi et al [14]	Case series

### Uniportal Versus Biportal Approaches

The two primary working approaches in endoscopic surgery include the uniportal and biportal techniques, each of which comes with advantages and drawbacks (Fig. 3C). The uniportal technique involves only one access site for both endoscope and working instruments, while biportal technique involves one access site for endoscope and a second site for the working instruments. Advantages of uniportal access include limited tissue disruption and simplicity, requiring fewer instruments [54]. Advantages of biportal access include improved visibility and continuous unidirectional saline irrigation [55]. Two recent meta-analyses comparing outcomes between uniportal and biportal techniques concluded that both methods were safe and reasonable for lumbar decompression, with comparable improvements in VAS and ODI, operative time, blood loss, and hospital stay [54, 56]. While there was no statistically significant difference in complication rate between the two, one meta-analysis showed a trend towards lower complication rate in biportal technique, presumably due to improved field of view [56].

### Complications and Complication Management

It is important to acknowledge the complications that can occur with endoscopic approaches. While the minimized tissue disruption and continuous irrigation provides advantages, the narrow working corridors and limited visibility can have significant drawbacks and require additional complication-management considerations. Firstly, dural tears represent the most common complication during endoscopic surgery, with reported rates ranging from 0% to 8.6% in the literature [57, 58]. Reasons for this include intermittently obstructed visibility, longer duration procedures with compounding risk of dural injury, continuous irrigation causing distortion or squeezing of dural edges which can then be damaged during ligamentum flavum removal, and smaller working corridors during bony drilling [59].

In uniportal endoscopy, the endoscope and operative instrument share a working corridor. In contrast, open and tubular microscopic techniques allow for simultaneous use of independent instruments, which enables surgeons to protect the dura with one instrument while removing bone with the other. While endoscopy removes the ability to retract the dura, the use of continuous saline irrigation facilitates hydrodissection between the ligamentum flavum and the dura [55]. This develops the epidural plane with minimal manipulation of the underlying dura. Control of irrigation pressure and maintaining an unobstructed fluid outflow are important as excessive hydrostatic pressure can lead to dural distortion.

There are many repair methods for dural tears, including local muscle and fat grafts, fibrin glue, and fibrin-collagen sponges such as TachoSil ^®^ (Corza Medical, Boulder, Colorado). Additionally, minimal muscle disruption may prevent symptomatic durotomy, as there is minimal corridor for continued postoperative leakage of cerebrospinal fluid [60, 61].

Symptomatic postoperative epidural fluid collection occurs rarely after endoscopic approach, but may occur more frequently after biportal technique [62]. Proposed explanations include the continuous irrigation, causing a buildup of epidural pressure, however lack of saline irrigation may negatively impact hemostasis, paradoxically increasing risk of epidural hematoma development [63]. Postoperative dysesthesia results from irritation of the exiting nerve root; particularly vulnerable during TED approaches. This complication can be mitigated by performing a foraminoplasty or enlarged foraminotomy to provide more room for the exiting nerve root [64]. The trans-SAP technique avoids blind entrance into the foramen as it produces an entrance corridor via the SAP [65]. Inadequate decompression or recurrent disc herniation can occur, particularly due to the limited field of view. Finally, postoperative infection rates after endoscopic surgery are low, even lower than traditional open techniques, thought to be due to the continuous saline irrigation and limited tissue retraction [60, 64, 66].

## Limitations and Controversies

Despite favorable clinical outcomes data and existence of techniques for several years, adoption of endoscopic techniques has not been ubiquitous. There are many explanations for the slower integration of endoscopy into spine surgery, one of which is the steep learning curve. One 2024 meta-analysis determined that the average case number to achieve proficiency in endoscopic techniques was 32.5 +/- 10.5 cases, more expedited in uniportal than biportal technique [68, 69]. Other retrospective reviews have found conflicting evidence regarding the comparative difficulty of biportal vs. uniportal technique, however it is largely agreed that both techniques involve this steep learning curve [70–72]. Predictably, novice surgeons required longer operative time and fluoroscopy time (FT). Additionally, complication rate was higher amongst novice surgeons than experienced surgeons, and VAS leg and back pain scores were improved amongst experienced operators [69]. Closely tied to this is the limited formalized training available to residents and practicing surgeons. Cadaver courses are a mainstay for practicing the technique prior to patient application, and apart from this, on-the-job learning and cases with occasional supervision from more experienced practitioners are common [73]. Virtual and augmented reality can be increasingly applied to training with novel techniques, endoscopy included [11]. In the future, formalized training pathways for those already in practice should be encouraged by institutions, and residency and fellowship curricula should prominently feature spinal endoscopic techniques. Operative time represents another important consideration in the adoption of endoscopic techniques. Procedures may initially be prolonged because of unfamiliar orientation, tissue manipulation and instrument exchange while working through a restricted corridor. However, operative time differences relative to open or tubular techniques are procedure-dependent. Endoscopic lumbar decompression and discectomy have comparable operative times to traditional approaches after surgeons overcome the learning curve [68]. In contrast, endoscopic lumbar interbody fusion consistently has longer operative times than traditional approaches, irrespective of surgeon experience [71]. Thus, prolonged operative time must not be considered a feature intrinsic to the field of endoscopic spine, but it remains particularly relevant during early adoption. This drawback should not be understated as prolonged operative time increases anesthesia exposure, surgeon fatigue, operating room utilization and direct procedural cost.

Another limiting factor is fluoroscopy time. C-arm is generally used for endoscopic localization and, compared to traditional open discectomy, is relied upon more throughout endoscopic discectomy and decompression due to limited view of surrounding anatomic landmarks. Transforaminal approaches have been shown to involve higher FT than interlaminar approaches (average 55 s versus 19 s, respectively) [74], and one study compared fluoroscopy usage between UBED, uniportal microdiscectomy, and MED, finding that FT and radiation exposure was highest in the uniportal group and lowest in the MED group [75]. As surgeon experience accumulates, so does FT, and, although higher than traditional open techniques, the radiation exposure to surgeon and patient still falls within safe limits.

Indications for endoscopic surgery are somewhat limited. Current evidence is strongest for focal pathology and limited multilevel decompression, commonly involving one to three operated motion segments (e.g. decompression at L3-4, L4-5, and L5-S1 is a three-level procedure). More extensive multilevel decompression may be feasible in appropriately selected patients, although the evidence supporting this is limited. Significant deformity requiring extensive correction or reconstruction remains less amenable to a purely endoscopic approach. Endoscopy can be employed in cervical, thoracic, and lumbar decompressions, and the biportal technique has increased visibility for cases with central canal stenosis and the need for more extensive decompression. Endoscopic TLIF is a newer technique gaining traction, with comparable outcomes to open TLIF and MIS-TLIF [33, 34]. However, in cases of bilateral foraminal stenosis, severe central canal stenosis, or significant deformity requiring correction, endoscopic TLIF is currently not advised for novice endoscopic surgeons. Additionally, recent data has shown the safety and efficacy of endoscopic techniques for revision lumbar discectomy and decompression, with complication rates comparable to traditional open techniques [76].

Lastly, there are cost and reimbursement challenges associated with widespread adoption of endoscopic surgery. Upfront costs related to endoscopic equipment are estimated at $350,000, which may be a barrier to initial hospital-system support [11]. One study compared costs related to open versus endoscopic lumbar decompression, and found that overall hospital costs were higher in the endoscopic group, primarily due to the high amount of disposable endoscopic supplies [77]. The authors did note that reduced length of stay, complication rates, and readmissions in the endoscopic group must be factored into long-term cost analyses to ensure a complete picture of the cost comparison. Despite higher initial surgical costs of TED compared to traditional open approaches, factors such as shorter length of stay and more rapid return to work has demonstrated possible societal cost savings in the long run, however this delayed impact may prove difficult to leverage against the aforementioned capital expenditure [78]. Insurance reimbursement also lags behind clinical data, with one main Current Procedural Terminology (CPT) code used (62380), despite widening indications and variable pathologies and levels of complexity associated with endoscopic approaches [11].

## Conclusion

Endoscopic spine surgery has transitioned from a niche technique to a comprehensive surgical platform with expanding indications. While current evidence supports its safety and efficacy in carefully selected patients, ongoing challenges, including training standardization, cost considerations, and technological integration, must be addressed. Future advancements in robotics, artificial intelligence, and minimally invasive fusion techniques are poised to further redefine the role of endoscopy in spine surgery.

## Key Reference

- Kim M, et al. Evolution of Spinal Endoscopic Surgery. Neurospine. 2019 Mar;16(1):6-14. https://doi.org/10.14245/ns.1836322.161. Epub 2019 Mar 31. PMID: 31618807; PMCID: PMC6449828.
  - ○ This landmark review was selected for its comprehensive description of the historical development and technical evolution of endoscopic spine surgery, providing important context for contemporary advances in the field.
- Antonacci CL, et al. A narrative review of endoscopic spine surgery: history, indications, uses, and future directions. J Spine Surg. 2024 Jun 21;10(2):295–304. https://doi.org/10.21037/jss-23-112. Epub 2024 May 10. PMID: 38974485; PMCID: PMC11224785.
  - ○ This contemporary review was selected for its broad synthesis of the expanding indications, current applications, and anticipated future directions of endoscopic spine surgery.
- Burkett D, Brooks N. Advances and Challenges of Endoscopic Spine Surgery. J Clin Med. 2024 Mar 1;13(5):1439. https://doi.org/10.3390/jcm13051439. PMID: 38592293; PMCID: PMC10932008.
  - ○ This article was selected for its balanced assessment of recent technological and procedural advances in endoscopic spine surgery alongside the challenges that continue to affect its adoption and implementation.
- Yong JH, et al. Full-endoscopic versus microscopic lumbar discectomy for lumbar disc herniation: a systematic review and meta-analysis of 4,186 cases. The Spine Journal. 2026 May 1;26(5):981–95. https://doi.org/10.1016/J.SPINEE.2025.12.016 . PubMed PMID: 41512930.
  - ○ This large contemporary meta-analysis was selected for its high-level comparative evidence evaluating the clinical effectiveness and perioperative outcomes of full-endoscopic versus conventional microscopic lumbar discectomy.
- Hu X, et al. Endoscopic Lumbar Interbody Fusion, Minimally Invasive Transforaminal Lumbar Interbody Fusion, and Open Transforaminal Lumbar Interbody Fusion for the Treatment of Lumbar Degenerative Diseases: A Systematic Review and Network Meta-Analysis. Global Spine J. 2024 Jan;14(1):295–305. https://doi.org/10.1177/21925682231168577. Epub 2023 Mar 31.
  - ○ This network meta-analysis was selected for its comparative evaluation of endoscopic lumbar interbody fusion against established minimally invasive and open fusion techniques, highlighting the expanding role of endoscopy beyond decompression and discectomy.
- Álvarez de Mon-Montoliú J, et al. Meta-Analysis of Learning Curve in Endoscopic Spinal Surgery: Impact on Surgical Outcomes. Global Spine J. 2024 May 1;15(4):2500. https://doi.org/10.1177/21925682241307634. PubMed PMID: 39637434.
  - ○ This meta-analysis was selected for its systematic characterization of the endoscopic spine surgery learning curve and its relationship to operative performance and clinical outcomes, addressing an important barrier to broader adoption of the technique.

## Data Availability

No datasets were generated or analysed during the current study.

## References

[1] Ravindra VM, et al. Degenerative Lumbar Spine Disease: Estimating Global Incidence and Worldwide Volume. Global Spine J. 2018;8(8):784–94. 10.1177/2192568218770769 . Epub 2018 Apr 24. PMC6293435 30560029

[2] Brinjikji W, et al. Systematic literature review of imaging features of spinal degeneration in asymptomatic populations. AJNR Am J Neuroradiol. 2015;36(4):811–6. 10.3174/ajnr.A4173 . Epub 2014 Nov 27. PMC4464797 25430861

[3] Park SM, Kim HJ, Yeom JS. Is minimally invasive surgery a game changer in spinal surgery? Asian Spine J. 2024;18(5):743–52. 10.31616/asj.2024.0337 . Epub 2024 Oct 22. PMC11538812 39434232

[4] Liounakos JI, et al. Intraoperative image guidance for endoscopic spine surgery. Ann Transl Med. 2021;9(1):92. 10.21037/atm-20-1119. PMC7859816 33553385

[5] Kim M, et al. Evolution of Spinal Endoscopic Surgery. Neurospine. 2019;16(1):6–14. 10.14245/ns.1836322.161 . Epub 2019 Mar 31. PMID: 31618807; PMCID: PMC6449828. PMC6449828 31618807

[6] Mayer HM. A History of Endoscopic Lumbar Spine Surgery: What Have We Learnt? Biomed Res Int. 2019;2019:4583943. 10.1155/2019/4583943 . PMID: 31139642; PMCID: PMC6470418. PMC6470418 31139642

[7] Ahn Y, A Historical Review of Endoscopic Spinal Discectomy. World Neurosurg. 2021;145:591–6. 10.1016/j.wneu.2020.08.008 . Epub 2020 Aug 8. PMID: 32781148. 32781148

[8] Wu PH, Kim HS, Jang IT. A Narrative Review of Development of Full-Endoscopic Lumbar Spine Surgery. Neurospine. 2020;17(Suppl 1):S20–33. 10.14245/ns.2040116.058 . Epub 2020 Jul 31. PMID: 32746515; PMCID: PMC7410380. PMC7410380 32746515

[9] Khandge AV, Sharma SB, Kim JS. The Evolution of Transforaminal Endoscopic Spine Surgery. World Neurosurg. 2021;145:643–56. 10.1016/j.wneu.2020.08.096 . Epub 2020 Aug 19. PMID: 32822954. 32822954

[10] Antonacci CL, et al. A narrative review of endoscopic spine surgery: history, indications, uses, and future directions. J Spine Surg. 2024;10(2):295–304. 10.21037/jss-23-112 . Epub 2024 May 10. PMID: 38974485; PMCID: PMC11224785. PMC11224785 38974485

[11] Burkett D, Brooks N. Advances and Challenges of Endoscopic Spine Surgery. J Clin Med. 2024;13(5):1439. 10.3390/jcm13051439 . PMID: 38592293; PMCID: PMC10932008. PMC10932008 38592293

[12] Huang CC, et al. Evolution of Cervical Endoscopic Spine Surgery: Current Progress and Future Directions-A Narrative Review. J Clin Med. 2024;13(7):2122. 10.3390/jcm13072122 . PMID: 38610887; PMCID: PMC11012719. PMC11012719 38610887

[13] Heo DH et al. History of endoscopic spine surgery: where did it all begin? Development of indications and techniques. Spine J. 2026;26(3):467–478. 10.1016/j.spinee.2025.07.031. Epub 2025 Jul 11. PMID: 40653021. 40653021

[14] Choi KC, et al. Comparison of Surgical Invasiveness Between Microdiscectomy and 3 Different Endoscopic Discectomy Techniques for Lumbar Disc Herniation. World Neurosurg. 2018;116:e750–8. 10.1016/j.wneu.2018.05.085 . Epub 2018 May 19. 29787880

[15] Yong JH, et al. Full-endoscopic versus microscopic lumbar discectomy for lumbar disc herniation: a systematic review and meta-analysis of 4,186 cases. Spine J. 2026;26(5):981–95. 10.1016/j.spinee.2025.12.016 . Epub 2026 Jan 7. 41512930

[16] Carr DA, Abecassis IJ, Hofstetter CP. Full endoscopic unilateral laminotomy for bilateral decompression of the cervical spine: surgical technique and early experience. J Spine Surg. 2020;6(2):447–56. 10.21037/jss.2020.01.03. PMC7340835 32656382

[17] Kwon WK, et al. Full Endoscopic Ligamentum Flavum Sparing Unilateral Laminotomy for Bilateral Recess Decompression: Surgical Technique and Clinical Results. Neurospine. 2022;19(4):1028–38. 10.14245/ns.2244344.172 . Epub 2022 Dec 31. PMC9816588 36597639

[18] Zhang J, et al. Comparison of percutaneous transforaminal endoscopic discectomy and open lumbar discectomy for lumbar disc herniations: A systematic review and meta-analysis. Front Surg. 2022;9:984868. 10.3389/fsurg.2022.984868. PMC9691761 36439526

[19] Heo DH, et al. Fully endoscopic lumbar interbody fusion using a percutaneous unilateral biportal endoscopic technique: technical note and preliminary clinical results. Neurosurg Focus. 2017;43(2):E8. 10.3171/2017.5.FOCUS17146 28760038

[20] Sofoluke N, et al. Full endoscopic resection of ventral thoracic osteophyte and repair of spontaneous CSF leak. Neurosurg Focus Video. 2024;10(2):V17. 10.3171/2024.1.FOCVID23209. PMC11013356 38616897

[21] Liles C, et al. Open Versus Endoscopic Approach for Thoracic Disk Herniations: Equivalent Short-Term Outcomes With Significantly Different Costs. Oper Neurosurg. 2025;28(3):347–56. 10.1227/ons.0000000000001325. 39189741

[22] Khan HA, et al. Thoracic endoscopic spine surgery: systematic review of the literature and exploring the margin of benefit. J Spine Surg. 2026;12(6):101. 10.21037/jss-2026-0065. PMC13351957 42434583

[23] Kim JE, et al. Biportal Endoscopic Spinal Surgery for Lumbar Spinal Stenosis. Asian Spine J. 2019;13(2):334–42. 10.31616/asj.2018.0210 . Epub 2019 Apr 30. PMC6454273 30959588

[24] Lobo K, et al. Uniportal Versus Biportal Endoscopic Decompression for the Treatment of Lumbar Spinal Stenosis: A Systematic Review and Updated Meta-Analysis. Global Spine J. 2025;15(7):3517–30. Epub 2025 Apr 29. 10.1177/21925682251339999 PMC12040859 40299717

[25] Perez-Roman RJ, et al. Endoscopic decompression for the treatment of lumbar spinal stenosis: an updated systematic review and meta-analysis. J Neurosurg Spine. 2021;36(4):549–57. 10.3171/2021.8.SPINE21890. 34767533

[26] Ahn Y. Endoscopic spine discectomy: indications and outcomes. Int Orthop. 2019;43(4):909–16. 10.1007/s00264-018-04283-w. Epub 2019 Jan 5. PMID: 30612170. 30612170

[27] Wang R, et al. Clinical Outcomes and Future Directions of Endoscopic Cervical Spine Surgery: A Systematic Review With Narrative Insights. Global Spine J. 2026;16(1suppl):S15–27. 10.1177/21925682251369437. Epub 2026 Jan 5. PMC12804027 41489665

[28] Casal Grau R, et al. Endoscopic Lateral Lumbar Interbody Fusion: Technical Note and Case Series. Int J Spine Surg. 2024;18(1):101–9. 10.14444/8572. PMC11265516 38320807

[29] Kotheeranurak V, et al. Complications in Full-Endoscopic Posterior Cervical Surgery: A Review of the Literature and Preventive Strategies. Global Spine J. 2025;15(7):3379–94. 10.1177/21925682251328615 . Epub 2025 Mar 25. PMC11948246 40131240

[30] Leyendecker J, et al. Full-endoscopic spine-surgery in the elderly and patients with comorbidities. Sci Rep. 2024;14(1):29188. 10.1038/s41598-024-80235-2. PMC11589573 39587174

[31] Sugiura K, et al. Transforaminal Full-Endoscopic Surgery for Lumbar Foraminal Pathologies: A Comparative Clinical Effectiveness Study. Neurosurgery. 2025;96(3S):S51–62. 10.1227/neu.0000000000003337 . Epub 2025 Feb 14. 39950784

[32] Prasse T, et al. Remote patient monitoring following full endoscopic spine surgery: feasibility and patient satisfaction. J Neurosurg Spine. 2023;39(1):122–31. 10.3171/2023.2.SPINE23136. 37060314

[33] Hu X, et al. Endoscopic Lumbar Interbody Fusion, Minimally Invasive Transforaminal Lumbar Interbody Fusion, and Open Transforaminal Lumbar Interbody Fusion for the Treatment of Lumbar Degenerative Diseases: A Systematic Review and Network Meta-Analysis. Global Spine J. 2024;14(1):295–305. Epub 2023 Mar 31. 10.1177/21925682231168577 PMC10676174 36999647

[34] Kou Y, et al. Endoscopic Lumbar Interbody Fusion and Minimally Invasive Transforaminal Lumbar Interbody Fusion for the Treatment of Lumbar Degenerative Diseases: A Systematic Review and Meta-Analysis. World Neurosurg. 2021;152:e352–68. 10.1016/j.wneu.2021.05.109 . Epub 2021 Jun 1. 34087465

[35] Stone BK, et al. Development of an Endoscopic Spine Surgery Program: Overview and Basic Considerations for Implementation. JB JS Open Access. 2023;8(3):e2200152. 10.2106/JBJS.OA.22.00152 PMC10508372 37731772

[36] Bani AA, Köhlert K, Zelenka M. Feasibility and safety of outpatient spine surgery: insights from 555 neurosurgical interventions. Eur Spine J. 2025 Aug;14. 10.1007/s00586-025-09257-2 . Epub ahead of print. 40804503

[37] Basil GW, Wang MY. Trends in outpatient minimally invasive spine surgery. J Spine Surg. 2019;5(Suppl 1):S108–14. 10.21037/jss.2019.04.17 PMC6626760 31380499

[38] Kilaru AS, et al. Savings Associated With Bundled Payments for Outpatient Spine Surgery Among Medicare Beneficiaries. JAMA Health Forum. 2025;6(7):e251907. 10.1001/jamahealthforum.2025.1907 PMC12254892 40643923

[39] Bohlen P, et al. The first week matters: App-based PROM trajectories and follow-up retention after endoscopic lumbar surgery. Brain Spine. 2026;6:106080. 10.1016/j.bas.2026.106080. PMC13188115 42170383

[40] Del Toro Runzer C, Balmayor ER, van Griensven M. Biologics for bone regeneration: advances in cell, protein, gene, and mRNA therapies. Bone Res. 2026;14(1):5. 10.1038/s41413-025-00487-0 PMC12791149 41521187

[41] Bonsu JM, et al. Orthobiologics in spine surgery: a narrative review. J Spine Surg. 2025;11(4):1065–72. 10.21037/jss-25-69 . Epub 2025 Dec 11. PMC12775640 41509834

[42] Bunch KM, et al. The symbiosis of robotics, enabling technology and minimally invasive surgery. N Am Spine Soc J. 2025;23:100769. 10.1016/j.xnsj.2025.100769 PMC12362081 40837070

[43] Younus I et al. Augmented Reality Assisted Spine Surgery: Applications and Future Directions. Oper Neurosurg. 2026 Jan 15. 10.1227/ons.0000000000001907 41537660

[44] Lee NJ, Lombardi JM, Lehman RA. Artificial Intelligence and Machine Learning Applications in Spine Surgery. Int J Spine Surg. 2023;17(S1):S18–25. 10.14444/8503 . Epub 2023 May 16. PMC10318911 37193608

[45] Liu S, et al. Comparing mid-term outcomes and patient satisfaction between percutaneous endoscopic lumbar discectomy and microendoscopic discectomy for foraminal and extraforaminal lumbar disc herniations: a retrospective matched cohort study. Front Surg. 2025;12:1554970. 10.3389/FSURG.2025.1554970/FULL. PMC11880218 40046499

[46] Tornatore I, et al. Effectiveness and Safety of Transforaminal Spinal Endoscopy: Analysis of 1000 Clinical Cases. Diagnostics. 2025;15(8):1021. 10. 3390/DIAGNOSTICS15081021 PubMed PMID: 40310433. 10.3390/diagnostics15081021 PMC12025548 40310433

[47] Wang S, et al. Percutaneous Endoscopic Lumbar Decompression for Lumbar Lateral Recess Stenosis: A Systematic Review. J Invest Surg. 2026;39(1). 10.1080/08941939.2026.2624189. 41672434

[48] Rajadurai J, et al. Comparison of outcomes in open and full endoscopic lumbar discectomies for treating lumbar radiculopathy in an Australian cohort. J Spine Surg. 2025;11(1):24–32. 10.21037/JSS-24-116/PRF. PMC11998043 40242811

[49] Li WS, Yan Q, Cong L. Comparison of Endoscopic Discectomy Versus Non-Endoscopic Discectomy for Symptomatic Lumbar Disc Herniation: A Systematic Review and Meta-Analysis. Global Spine J. 2022;12(5):1012–26. doi:10.1177/21925682211020696;PAGE:STRING:ARTICLE/CHAPTER. 10.1177/21925682211020696 PMC9344526 34402320

[50] Patel T, et al. Comparative effectiveness of minimally invasive endoscopic discectomy versus conventional surgical techniques for lumbar disc herniation: a systematic review and meta-analysis. Annals Med Surg. 2025;87(10):6661. 10.1097. /MS9.0000000000003689 PubMed PMID: 41181429. 10.1097/MS9.0000000000003689 PMC12577904 41181429

[51] Derman PB, et al. Return to work after lumbar endoscopic spinal surgery in the United States. Spine J. 2025;25(12):2728–38. 10.1016/j.spinee.2025.05.033 . Epub 2025 May 24. 40418992

[52] Ward AJ, et al. Comparison of endoscopic and non-endoscopic lumbar decompression outcomes using ACS-NSQIP database 2017–2022. J Spine Surg. 2025;11(2):234–41. 10.21037/JSS-24-163. /COIF PubMed PMID: 40621378. PMC12226183 40621378

[53] Leyendecker J, et al. Full-endoscopic spinal decompression or discectomy show benefits regarding 30-day readmission rates when compared to other spine surgery techniques: a propensity score matched analysis. Spine J. 2025;25(5):996–1005. Epub 2024 Dec 3. 10.1016/j.spinee.2024.11.007 39631464

[54] Singh S, et al. Comparison of Biportal and Uniportal Spine Endoscopy for Effectiveness and Perioperative Safety: A Systematic Review and Meta-Analysis. Cureus. 2025;17(12):e98764. 10.7759/CUREUS. .98764 PubMed PMID: 41523539. PMC12779580 41523539

[55] Choi CM. Biportal endoscopic spine surgery (BESS): considering merits and pitfalls. J Spine Surg. 2020;6(2):457. 10.21037/JSS. 2019.09.29 PubMed PMID: 32656383. PMC7340825 32656383

[56] Xu W, Bin, et al. Is Biportal Endoscopic Spine Surgery More Advantageous Than Uniportal for the Treatment of Lumbar Degenerative Disease? A Meta-Analysis. Med (B Aires). 2022;58(11):1523. 10. 3390/MEDICINA58111523 PubMed PMID: 36363480. 10.3390/medicina58111523 PMC9698221 36363480

[57] Müller SJ, Burkhardt BW, Oertel JM. Management of Dural Tears in Endoscopic Lumbar Spinal Surgery: A Review of the Literature. World Neurosurg. 2018;119:494–9. .2018.05.251 PubMed PMID: 29902608. 10.1016/j.wneu.2018.05.251 29902608

[58] Greil ME, et al. Full-endoscopic trans-pars interarticularis approach for far lateral lumbar discectomy. Eur Spine J. 2023;32(8):2709–16. 10.1007/s00586-023-07698-1 . Epub 2023 May 11. 37166550

[59] Ju C, Il, Lee SM. Complications and Management of Endoscopic Spinal Surgery. Neurospine. 2023;20(1):56–77. 10.14245/NS.2346226.113. PMC10080410 37016854

[60] Klingler JH, et al. Accidental Durotomy in Minimally Invasive Transforaminal Lumbar Interbody Fusion: Frequency, Risk Factors, and Management. Sci World J. 2015;2015(1):532628. 10.1155/2015/532628 . PubMed PMID: 26075294. PMC4449940 26075294

[61] Ahn DK, et al. Postoperative spinal epidural hematoma in a biportal endoscopic spine surgery. Med (United States). 2021;100(6):E24685. 10. 1097/MD.0000000000024685 PubMed PMID: 33578600. 10.1097/MD.0000000000024685 PMC10545396 33578600

[62] Kang MS, et al. Clinical outcome of biportal endoscopic revisional lumbar discectomy for recurrent lumbar disc herniation. J Orthop Surg Res 2020. 2020;15(1):1. 10.1186/S13018-020-02087-6 . PubMed PMID: 33228753. PMC7685633 33228753

[63] Fan N, et al. Complications and risk factors of percutaneous endoscopic transforaminal discectomy in the treatment of lumbar spinal stenosis. BMC Musculoskelet Disorders 2021. 2021;22(1):1. 10.1186/S12891-021-. 04940-Z PubMed PMID: 34911532. PMC8672468 34911532

[64] Mahan MA, et al. Full-endoscopic spine surgery diminishes surgical site infections – a propensity score-matched analysis. Spine J. 2023;23(5):695–702. 10.1016/j.spinee.2023.01. .009 PubMed PMID: 36708928. 36708928

[65] Hasan S, Härtl R, Hofstetter CP. The benefit zone of full-endoscopic spine surgery. J Spine Surg. 2019;5(Suppl 1):S41–56. 10.21037/jss.2019.04.19. PMC6626753 31380492

[66] Bucknall V, Gibson JNA. Cervical endoscopic spinal surgery: A review of the current literature. J Orthop Surg. 2018;26(1). 10.1177/2309499018758520 29455630

[67] Ruetten S, et al. Full-endoscopic cervical posterior foraminotomy for the operation of lateral disc herniations using 5.9-mm endoscopes: a prospective, randomized, controlled study. Spine (Phila Pa 1976). 2008;33(9):940–8. 10.1097/BRS.0B013. E31816C8B67 PubMed PMID: 18427313. 18427313

[68] Álvarez de Mon-Montoliú J, et al. Meta-Analysis of Learning Curve in Endoscopic Spinal Surgery: Impact on Surgical Outcomes. Global Spine J. 2024;15(4):2500. doi:10.1177/21925682241307634 PubMed PMID: 39637434. 10.1177/21925682241307634 PMC11622208 39637434

[69] Perfetti DC, et al. Learning curve for endoscopic posterior cervical foraminotomy. Eur Spine J. 2023;32(8):2670–8. 10.1007/s00586-023-07623-6 . Epub 2023 Mar 3. 36867253

[70] Wu PH, Kim HS, Choi DJ, Gamaliel YHT. Overview of Tips in Overcoming Learning Curve in Uniportal and Biportal Endoscopic Spine Surgery. J Minim Invasive Spine Surg Tech. 2021;6(Suppl 1):S84-S96. Publication date (electronic): 2021 April 29 10.21182/jmisst.2020.00024

[71] Liu Y, et al. Learning Curve of Uniportal Compared With Biportal Endoscopic Techniques for the Treatment of Lumbar Disc Herniation. Orthop Surg. 2025;17(2):513–24. 10.1111/os.14312 . Epub 2024 Dec 13. PMID: 39673164; PMCID: PMC11787971. PMC11787971 39673164

[72] Alostaz M, et al. Attitudes regarding barriers to entry and the learning curve associated with endoscopic decompression-only surgery: an international survey. Spine J. 2025;25(5):983–95. Epub 2024 Nov 27. 10.1016/j.spinee.2024.11.009 39613036

[73] Chung AS, et al. Endoscopic spine surgery—increasing usage and prominence in mainstream spine surgery and spine societies. J Spine Surg. 2020;6(Suppl 1):S14. 10.21037/JSS. 2019.09.16 PubMed PMID: 32195409. PMC7063299 32195409

[74] Tsipouriaris P, et al. Intra-operative fluoroscopy exposure in endoscopic lumbar discectomies. Surg Neurol Int. 2025;16:305. doi:10.25259/SNI_523_2025 PubMed PMID: 40837308. 10.25259/SNI_523_2025 PMC12361666 40837308

[75] Merter A, Karaeminogullari O, Shibayama M. Comparison of Radiation Exposure Among 3 Different Endoscopic Diskectomy Techniques for Lumbar Disk Herniation. World Neurosurg. 2020;139:e572–9. .2020.04.079 PubMed PMID: 32330613. 10.1016/j.wneu.2020.04.079 32330613

[76] Glauser et al. Revision Status and Patient-Reported Outcomes in Thoracolumbar Endoscopic Spine Surgery: A Multi-Center Cohort Study. Neurosurg Focus. *In press*.

[77] Findlay MC, et al. Hospital cost differences between open and endoscopic lumbar spine decompression surgery. J Neurosurg Spine. 2023;40(1):77–83. 10.3171/2023.8. .SPINE23439 PubMed PMID: 37856388. 37856388

[78] Gadjradj PS, et al. Cost-effectiveness of full endoscopic versus open discectomy for sciatica. Br J Sports Med. 2022;56(18):1018. 10. 1136/BJSPORTS-2021-104808 PubMed PMID: 35185010. 10.1136/bjsports-2021-104808 PMC9484367 35185010

